# Self-compensation in chlorine-doped CdTe

**DOI:** 10.1038/s41598-019-45625-x

**Published:** 2019-06-24

**Authors:** Walter Orellana, Eduardo Menéndez-Proupin, Mauricio A. Flores

**Affiliations:** 1 Departamento de Ciencias Físicas, Universidad Andres Bello, Sazié 2212, Santiago, 0370136 Chile; 2 Departamento de Física, Facultad de Ciencias, Universidad de Chile, Las Palmeras 3425, Ñuñoa, Santiago, 7800003 Chile; 3 Facultad de Ingeniería y Tecnología, Universidad San Sebastián, Bellavista 7, Santiago, 8420524 Chile

**Keywords:** Semiconductors, Electronic properties and materials

## Abstract

Defect energetics, charge transition levels, and electronic band structures of several Cl-related complexes in CdTe are studied using density-functional theory calculations. We investigate substitutional chlorine (Cl_Te_ and Cl_Cd_) and complexes formed by Cl_Te_ with the cadmium vacancy (Cl_Te_-V_Cd_ and 2Cl_Te_-V_Cd_) and the Te_Cd_ antisite (Cl_Te_-Te_Cd_). Our calculations show that none of the complexes studied induce deep levels in the CdTe band gap. Moreover, we find that Cl_Te_-V_Cd_ and Cl_Te_ are the most stable Cl-related centers in *n*-type and *p*-type CdTe, under Te-rich growth conditions, showing shallow donor and acceptor properties, respectively. This result suggests that the experimentally-observed Fermi level pinning near midgap would be originated in self-compensation. We also find that the formation of the Cl_Te_-Te_Cd_ complex passivates the deep level associated to the Te antisite in neutral charge state.

## Introduction

Cadmium telluride (CdTe) is a II-VI chalcogenide semiconductor with a band gap energy of ∼1.5 eV at room temperature, being one of the few that can be relatively easily doped *p*- and *n*-type. CdTe has been actively investigated for more than 70 years for photovoltaics^1–3^, room-temperature *γ*- and x-ray radiation detection^4–7^, as well as medical imaging^8^. Due to its high stability (formation enthalpy ∼100 kJ mol^−1^), high absorption coefficient (>5 × 10^5^ cm^−1^), and near optimum band gap for visible absorption, CdTe is very suitable material for use as absorber layer in thin-film solar cells. In fact, single-junction CdTe-based solar cells have recently reached conversion efficiencies as high as 22.1%^9^. Additionally, its low manufacturing cost and the continuing improvement on its conversion efficiency have positioned CdTe among one of the most promising materials alternative to crystalline silicon, which dominates the global photovoltaic market^10^.

In addition, the large CdTe band gap allows its use in *γ*- and x-ray detectors, operating at room temperature with modest cooling^11^, as opposed to germanium-based detectors that require cryogenic temperatures to operate. Moreover, high-quality radiation detectors require an intrinsic high resistivity to reduce dark currents, a high mobility-lifetime product of electrons (*μτ*), and a high atomic weight^12^. The former property can be achieved in CdTe by self-compensation of intrinsic point defects during the crystal growth, while the latter are guaranteed by its good charge transport properties and large atomic number of its constituents.

It is widely known that native defects usually play an important role by limiting the *p*- or *n*-type conductivity in a semiconductor^13–17^, which may be achieved by extrinsic doping^18,19^. Due to its importance and the complexity of the problem, understanding the effect of self-compensation in CdTe is a major focus of current research in the field^15,20–23^. Moreover, in the case of CdTe, group IV elements can be used as extrinsic dopants to achieve a semi-insulating material^24–27^. However, these impurities normally introduce deep levels in the band gap, which may act as non-radiative Shockley-Read-Hall recombination centers^28,29^ with a deleterious effect on the performance of the detector. This does not apply to chlorine doping of CdTe, which results in a high-resistive material with good carrier transport properties^30,31^, suitable for *γ*- and x-radiation detection^32,33^; still, the origin of its electric properties is not well understood and remains as an open issue^34^.

The dominant compensation model for detector-grade CdTe:Cl is based on the existence of one compensating deep level located at ∼0.725 eV above the valence band maximum (VBM)^12,34^, which might be responsible for the Fermi level pinning near midgap. This model is supported by experimental observations, but it cannot be explained by theory. In this context, several theoretical works have suggested that compensation between shallow donors and acceptors, rather than one deep level, should be responsible for the Fermi level pinning in CdTe:Cl^35,36^. However, other works have concluded that high-resistivity in CdTe:Cl cannot be explained by shallow defect levels alone^37^.

In this work, we set out to determine the origin of the high-resistivity experimentally observed in detector-grade CdTe:Cl. In that aim, we calculate from ab-initio the formation energies, electronic structure, and transition states of three chlorine-related defects in CdTe: (i) the isolated substitutional chlorine, that is Cl occupying a Te site (Cl_Te_) and a Cd site (Cl_Cd_); (ii) the two complexes formed by Cl_Te_ nearest neighbor to the cadmium vacancy (V_Cd_). The first one contains one Cl_Te_ neighboring to the vacancy (Cl_Te_-V_Cd_), which is commonly known as the A-center, while the second one contains two Cl_Te_ neighboring to the vacancy (2Cl_Te_-V_Cd_); and (iii) the complex formed by the Cl_Te_ impurity nearest neighbor to the Te_Cd_ antisite (Cl_Te_-Te_Cd_). Our results show that Cl_Te_-V_Cd_ is the most stable Cl-related complex in *n*-type CdTe, that together with Cl_Te_, the most stable Cl impurity in *p*-type CdTe, would play a key role in the self-compensation mechanism. Indeed, our results support the hypothesis that compensation between shallow donors and acceptors alone can explain the good electron mobility and high-resistivity of CdTe:Cl, in agreement with experimental observations.

## Results and Discussion

### Substitutional chlorine in CdTe

**Figure 1:**
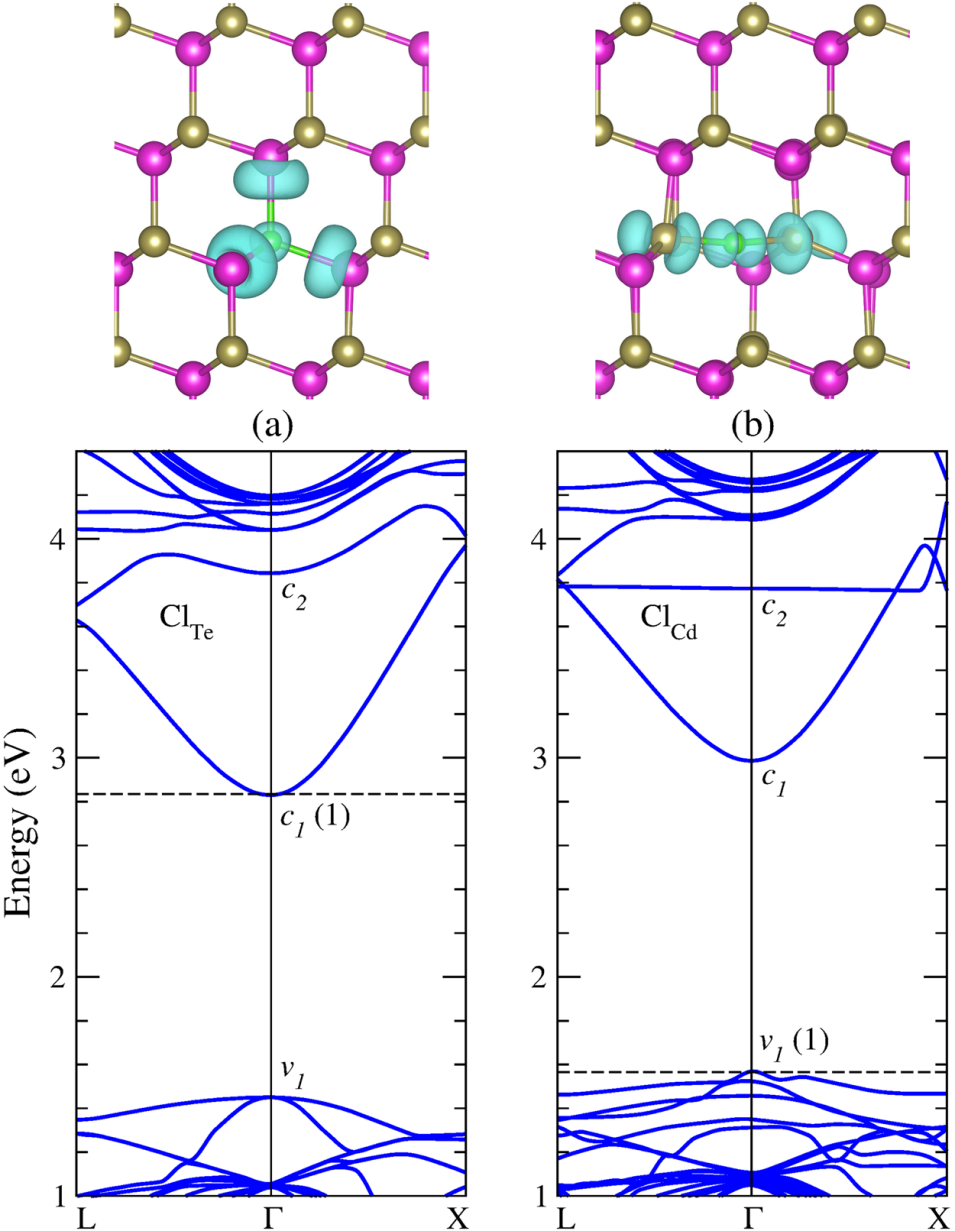
Equilibrium geometry and band structure calculations of substitutional chlorine in the neutral charge state. (**a**) Cl_Te_, and (**b**) Cl_Cd_. The dashed line indicates the Fermi energy. Charge density isosurface of the impurity level (*c*_2_) of Cl_Te_ and Cl_Cd_ are plotted for *ρ* = 0.001 and 0.005 *e*/Å^3^, respectively. Violet, brown, and green balls represent Cd, Te, and Cl atoms, respectively.

As an isolated substitutional impurity, chlorine can occupy only a Cd site or a Te site in CdTe. Our initial model was simply substitute a lattice atom with the neutral Cl impurity and left the system to relax. Figure 1(a) shows the equilibrium geometries and band structure of substitutional chlorine in CdTe in the neutral charge state. Our results for chlorine occupying a Te site (Cl_Te_) indicate a four-fold coordinated impurity with almost undistorted geometry, preserving the T_d_ symmetry. The Cd atoms next to the impurity relax outwards by about 3%, resulting in a Cd-Cl bond distance of 2.936 Å. The same configuration is observed in single positive charge state. The electronic properties indicate that Cl_Te_ is a shallow donor, introducing an impurity level resonant in the conduction band (*c*_2_). The squared wavefunction, or simply the charge density, corresponding to this level indicates a Cl-Cd antibonding character, as shown Fig. 1(a). The electronic structure shows a band gap of 1.40 eV, close to that calculated for pristine CdTe (1.44 eV).

Figure 1(b) shows the equilibrium geometries and band structure of chlorine occupying a Cd site (Cl_Cd_), revealing a strong relaxation from the T_d_ symmetry. In the neutral charge state, the impurity has two-fold coordination, forming a bridge-like structure between two Te atoms. The Te-Cl bond distances are found to be of 2.627 Å with a Te-Cl-Te bond angle of 169°. Whereas, the two other Te atoms become three-fold coordinated at a distance of 3.75 Å from the Cl atom. The same configuration is observed in single negative charge state. It is interesting to note that the Cl_Cd_ equilibrium configuration is similar to that expected for the V_Cd_-Cl_i_ complex. The electronic properties of Cl_Cd_ show that it is a shallow acceptor that introduces a strong perturbation on the top of the valence band. These states are associated with the three-fold coordinated Te atoms next to the Cl atom. In addition, an impurity level with an antibonding character (*c*_2_) is found to be resonant in the conduction band, as shown Fig. 1(b).

**Figure 2:**
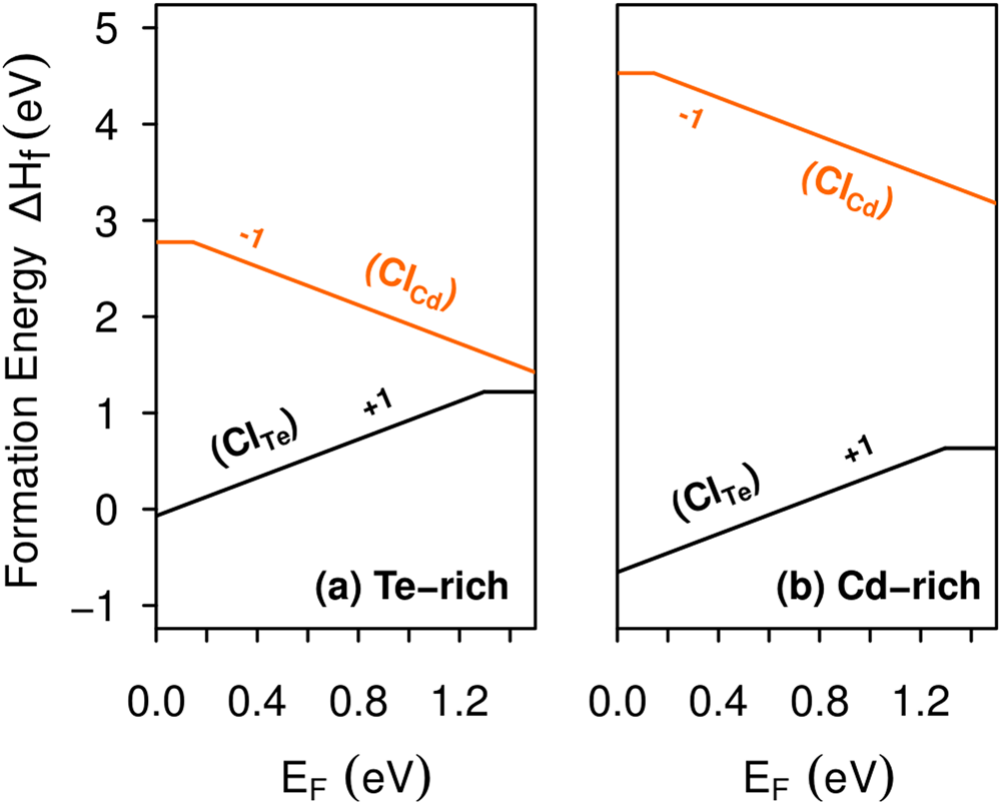
Formation energies of substitutional chlorine in CdTe, Cl_Te_ and Cl_Cd_, as a function of the Fermi level, under (**a**) Te-rich condition and (**b**) Cd-rich condition. The slopes of the formation energy lines indicate the charge states and changes in its slopes indicates defect transition states.

The stability of substitutional chlorine in CdTe is obtained through formation energy calculations, as shown in Fig. 2. Our results indicates that Cl_Te_ is the most stable impurity for both Cd-rich and Te-rich growth conditions. Moreover, the Cl_Te_ impurity is a shallow donor with a *ε*(+/0) transition level at VBM + 1.29 eV, while Cl_Cd_ is a shallow acceptor that introduces a *ε*(0/−) transition level at VBM + 0.15 eV. However, Cl_Cd_ shows a high formation energy in comparison with Cl_Te_, suggesting that it would be unlikely to form at relevant concentrations.

### The Cl_Te_-V_Cd_ complex in CdTe

The Cl_Te_-V_Cd_ complex in CdTe is formed when a Cl atom replaces a Te atom nearest neighbor to a Cd vacancy^38^. Previous DFT calculations have identified the isolated Cd vacancy as the dominant intrinsic acceptor in CdTe^39–41^. We found that V_Cd_ is stable in *T*_*d*_, *D*_2*d*_, and *C*_2*v*_ symmetries, being the V_Cd_(*C*_2*v*_) configuration the global minimum. We also found that V_Cd_ is stable in *C*_3*v*_ symmetry in the single-negative charge state, which is stabilized by a hole polaron, in good agreement with previous calculations^41^. In the neutral charge state, V_Cd_(*T*_*d*_) and V_Cd_(*D*_2*d*_) are 0.64 and 2.11 eV higher in energy than V_Cd_(*C*_2*v*_), respectively. The V_Cd_(*C*_2*v*_) structure is characterized by the formation of a Te-Te dimer between two undercoordinated Te atoms neighboring to the vacancy. Our results show that this dimer has a bond distance of 2.773 Å, while the others Te atoms remain three-fold coordinated and are displaced inward the vacancy by about 0.24 Å with respect to their perfect crystal positions. Interestingly, the V_Cd_(*C*_2*v*_) in the neutral charge state exhibits a ground-state configuration with all the valence band filled and all the conduction bands empty. This result is in close agreement with recent DFT-HSE06 calculations, although the formation of the Te-Te dimer was not reported^42^. On the other hand, the V_Cd_(*D*_2*d*_) structure reveals the formation of two Te-Te dimers with bond distances of 2.765 Å, while its electronic structure indicates a double donor character.

**Figure 3:**
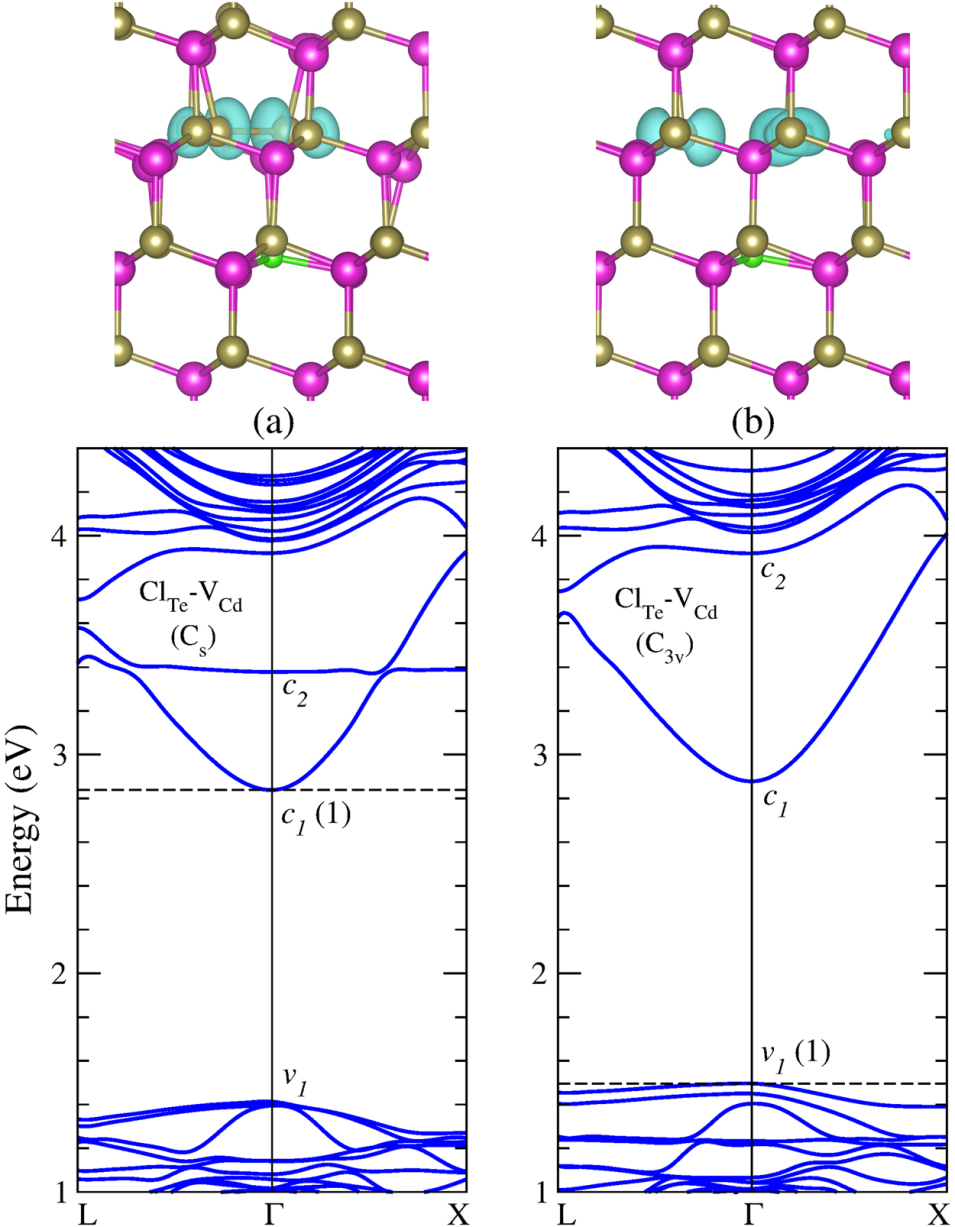
Equilibrium geometry and band structure calculations of the complexes (**a**) (Cl_Te_-V_Cd_) (*C*_*s*_), and (**b**) (Cl_Te_-V_Cd_)(*C*_3*v*_) in the neutral charge state. The dashed line indicates the Fermi energy. Charge density isosurfaces for the impurity level (*c*_2_) in (**a**) and the level at VBM (*v*_1_) in (**b**) are plotted for *ρ* = 0.005 *e*/Å^3^ and *ρ* = 0.001 *e*/Å^3^, respectively.

Next, we investigate the formation of the Cl_Te_-V_Cd_ complex starting from the V_Cd_(*T*_*d*_) geometry by substituting a neighboring Te atom by a Cl atom. Our results for the electronic band structure and the equilibrium geometry are shown in Fig. 3(b). We find that the neutral complex is stable in *C*_3*v*_ symmetry [hereafter referred as (Cl_Te_-V_Cd_)(*C*_3*v*_)], where the Cl_Te_ impurity moves outward the vacancy by 0.5 Å, while the three-fold coordinated Te atoms relax inward by 0.29 Å with respect to their perfect crystal positions, as shown in Fig. 3(b). Moreover, neutral (Cl_Te_-V_Cd_)(*C*_3*v*_) has a hole at the VBM, indicating a shallow acceptor character in good agreement with previous theoretical^35,43,44^ and experimental^32^ results.

By taking into account the high stability of the V_Cd_(*C*_2*v*_) structure that form the Te-Te dimer, we construct another complex by substituting an under-coordinated Te atom by a Cl atom. Our results for the band structure and equilibrium geometry of this complex are shown in Fig. 3(a). We find that the new complex geometry preserves the Te-Te dimer of the V_Cd_(*C*_2*v*_) configuration with a bond distance of 2.771 Å. Moreover, we found that the substitutional chlorine moves outward the Cd vacancy by 0.5 Å. This complex has *C*_*s*_ symmetry with only one symmetry plane [hereafter referred as (Cl_Te_-V_Cd_)(*C*_*s*_)], which is 0.73 eV higher in energy than the (Cl_Te_-V_Cd_)(*C*_3*v*_) structure in the neutral charge state. It is worth noting that the Cl_Te_-V_Cd_ complex was recently studied by Lindström *et al*., using DFT-HSE06 calculations^36^. They found two equilibrium geometries for this complex, which is stable in both *C*_*s*_ and *C*_3*v*_ symmetries, in the neutral and in the negative charge state, respectively. Although the two configurations of the complex agree with our calculations, the equilibrium geometry of the neutral (Cl_Te_-V_Cd_)(*C*_*s*_) differs from our results. We find the formation of a Te-Te dimer, while Lindström *et al*.^36^ found only a small approach between the same Te atoms, suggesting that its configuration may be a metastable state.

Our results shows that the (Cl_Te_-V_Cd_)(*C*_*s*_) complex has a shallow donor property, in contrast to the (Cl_Te_-V_Cd_)(*C*_3*v*_) complex, which is a shallow acceptor, as shown Fig. 3. Although with a higher formation energy, the (Cl_Te_-V_Cd_)(*C*_*s*_) complex is likely to form in the single-positive charge state, which is a ground state configuration with all the valence band filled and all the conduction bands empty. Indeed, for the Fermi energy close to the VBM the [(Cl_Te_-V_Cd_)(*C*_*s*_)]^+^ configuration has much lower energy than the [(Cl_Te_-V_Cd_)(*C*_3*v*_)]^−^ polaronic configuration found in ref.^36^, which acts as a harmful electron trap.

**Figure 4:**
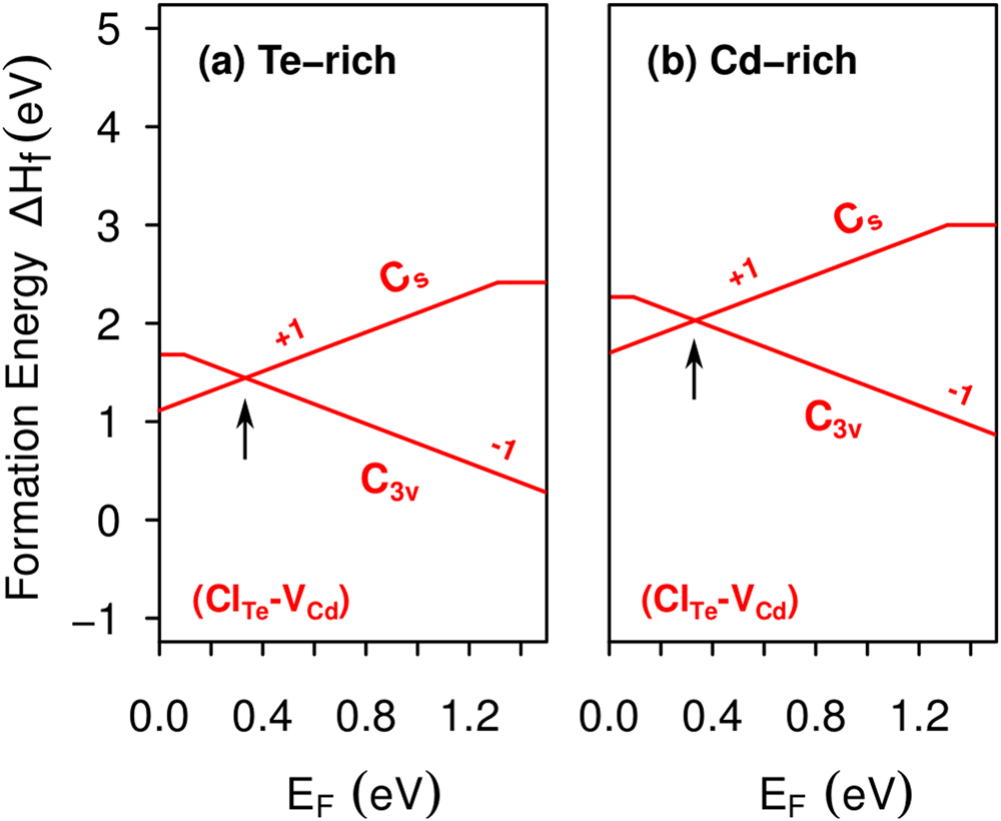
Formation energies of the Cl_Te_-V_Cd_ complex in *C*_*s*_ and *C*_3*v*_ symmetries, as a function of the Fermi level, under (**a**) Te-rich condition and (**b**) Cd-rich condition.

**Figure 5:**
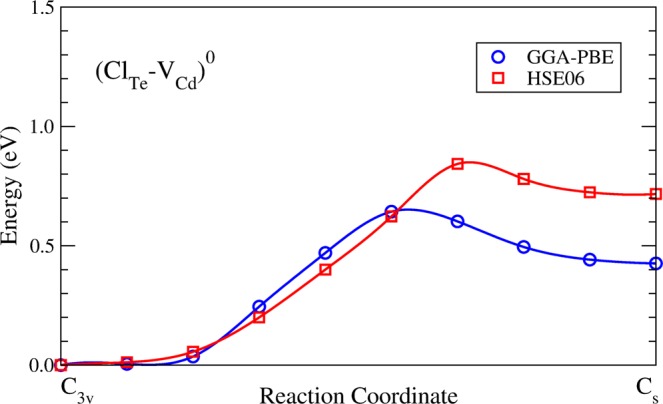
Minimum energy paths for transition of the neutral complex Cl_Te_-V_Cd_ from *C*_3*v*_ to *C*_*s*_ symmetries, as calculated with GGA-PBE and HSE06 functionals.

Figure 4 shows the formation energies of (Cl_Te_-V_Cd_)(*C*_*s*_) and (Cl_Te_-V_Cd_)(*C*_3*v*_), indicating that both complexes can coexist for the Fermi energy close to VBM + 0.33 eV, the energy at which their formation energies are equal, as indicated by the arrow. As the transition between the *C*_3*v*_ and *C*_*s*_ geometries involves the formation of a Te-Te dimer, we want to know the energy needed to overcome the barrier between them, that is the activation energy, and if this process is likely to occur at the operational conditions. To do that, we calculate the minimum energy path (MEP) of neutral Cl_Te_-V_Cd_ moving from *C*_3*v*_ to *C*_*s*_ geometries, using the climbing-image nudged elastic band (NEB) method^45^. This method finds the MEP between two local minima previously obtained, by optimizing intermediate geometries called images. Then, the activation energy is obtained by calculating the difference between the lowest minimum and the saddle point. In our calculations we obtain the MEP using GGA-PBE functional, considering a 128-atom supercell with all atoms free to relax, and ten images between the *C*_3*v*_ and *C*_*s*_ local minima. We obtain an activation energy of 0.64 eV, as shown in Fig. 5.

To estimate the MEP using the hybrid HSE06 functional, which is the functional used throughout this work, we re-evaluated the GGA-PBE images previously found using now the HSE06 functional without allow the system to relax (single-point energy). It is important to note that NEB calculations using directly the HSE06 functional are not possible due to the huge computational time required. Our results show an activation energy of 0.85 eV, as shown in Fig. 5. The difference between both minimum energy paths is partially attributed to a residual strain in the single-point calculation and the inclusion of the fraction of exact exchange due to the hybrid functional. It is interesting to note that the larger difference between MEPs is obtained when the Te-Te dimer becomes to form. We believe that a realistic activation energy for transition between the *C*_3*v*_ and *C*_*s*_ geometries should be something between GGA-PBE and HSE06 calculations. Experiments have measured activation energies for the chlorine diffusion in CdTe to be of 0.63 and 1.32 eV for a temperature range between 200 and 700 °C^46^. Therefore, the activation energy for the *C*_3*v*_ to *C*_*s*_ transition in the neutral Cl_Te_-V_Cd_ complex lies in the range of 0.64 to 0.85 eV, suggesting that it is likely to occur.

However, the activation energy for the inverse transition *C*_*s*_ to *C*_3*v*_, which would be the most probable direction, is just of 0.15 eV. Therefore, at the HSE06 level of calculation, the formation of the Cl_Te_-V_Cd_ complex would start with a neutral V_Cd_ vacancy with *C*_2*v*_ geometry. If a Cl impurity occupies the position of one of the two three-fold coordinated Te atoms surrounding the vacancy, a neutral (Cl_Te_-V_Cd_)(*C*_*s*_) complex would form. According to Fig. 3(a), this complex is a shallow donor being likely to lose an electron, changing to the (Cl_Te_-V_Cd_)(*C*_3*v*_) geometry after overcoming an energy barrier of 0.15 eV.

### The 2Cl_Te_-V_Cd_ complex in CdTe

**Figure 6:**
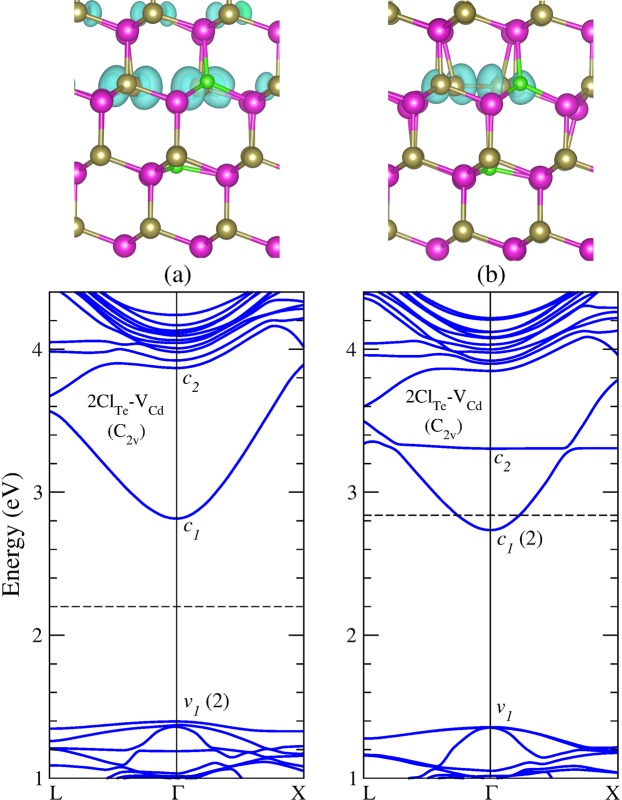
Equilibrium geometry and band structure calculations of the neutral 2Cl_Te_-V_Cd_ complex in the two stable configurations (**a**) with Te atoms separated, and (**b**) with the Te atoms forming a dimer. The dashed line indicates the Fermi energy. Charge density isosurfaces for the level at the VBM (*v*_1_) in (**a**) and the impurity level (*c*_2_) in (**b**) are plotted for *ρ* = 0.001 *e*/Å^3^ and *ρ* = 0.005 *e*/Å^3^, respectively.

We subsequently investigate the possibility of a new complex consisting of two Cl atoms substituting two of the four undercoordinated Te atoms nearest neighbors to a Cd vacancy (hereafter referred as 2Cl_Te_-V_Cd_). This complex was predicted in 1974 by Canali *et al*.^47^, suggesting that it would be likely to be found in high-resistivity donor-doped CdTe. After substituting the second Te atom by a Cl atom in (Cl_Te_-V_Cd_)(*C*_3*v*_) as shown in Fig. 3(b), we observe that the system relaxes to the 2Cl_Te_-V_Cd_ configuration shown in Fig. 6(a), exhibiting *C*_2*v*_ symmetry. The inclusion of the second chlorine fills the hole that (Cl_Te_-V_Cd_)(*C*_3*v*_), shows at the VBM, stabilizing the (2Cl_Te_-V_Cd_)(*C*_2*v*_) complex in neutral charge state. Moreover, the electronic structure of this complex shows that the neutral charge state is a ground-state configuration, that is with all the valence bands filled and all the conduction bands empty.

In addition, we find a second configuration for the 2Cl_Te_-V_Cd_ complex, where the two adjacent undercoordinated Te atoms form a Te-Te dimer with a bond length of 2.771 Å, preserving the *C*_2*v*_ symmetry. In the neutral charge state, this complex has two excess electrons in the conduction bands, as shown in Fig. 6(b), indicating that 2Cl_Te_-V_Cd_ is a double donor that would tend to transfer its excess electrons to uncompensated acceptors such as the V_Cd_. In this way, the ground-state configuration [2Cl_Te_-V_Cd_]^2+^ should be stabilized.

**Figure 7:**
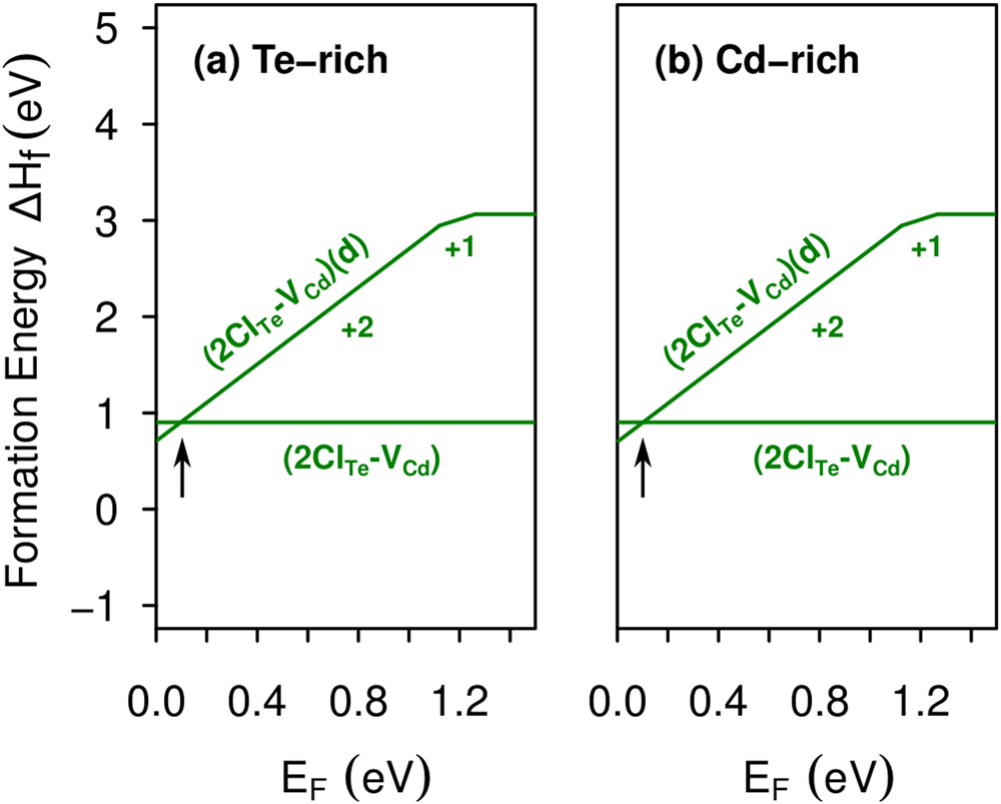
Formation energies of the 2Cl_Te_-V_Cd_ complex as a function of the Fermi level, under (**a**) Te-rich condition and (**b**) Cd-rich condition. The neutral charge state represents the complex with separated Te atoms and the positive charge states represent the complex with a Te dimer.

Figure 7 shows the calculated formation energies for the two configurations of the 2Cl_Te_-V_Cd_ complex as a function of the Fermi level, under both Te-rich and Cd-rich growth conditions. We find that the Te-Te dimer configuration, which will be referred as (2Cl_Te_-V_Cd_)(*d*), has the lowest formation energy for the Fermi level close to the VBM (*n*-type CdTe), being likely to be found under this condition. Whereas for other Fermi level positions, the complex configuration with the separated Te atoms is the most stable. Thus, the crossing point of formation energy lines, indicated by arrows in Fig. 7, shows that 2Cl_Te_-V_Cd_ introduces a shallow transition level *ε*(2 + /0) at VBM + 0.1 eV. Interestingly, this complex exhibits the same defect formation energies under both Te-rich and Cd-rich growth conditions.

### The Cl_Te_-Te_Cd_ complex in CdTe

**Figure 8:**
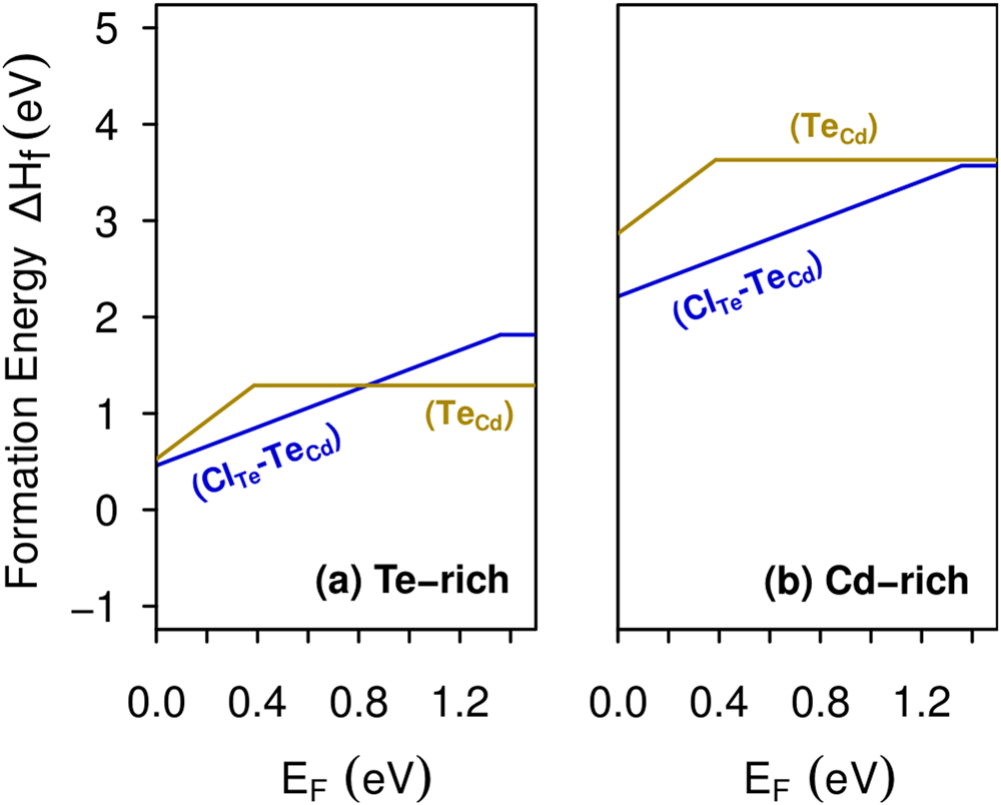
Formation energies of the Te_Cd_ antisite and the Cl_Te_-Te_Cd_ complex as a function of the Fermi level, under (**a**) Te-rich condition and (**b**) Cd-rich condition.

**Figure 9:**
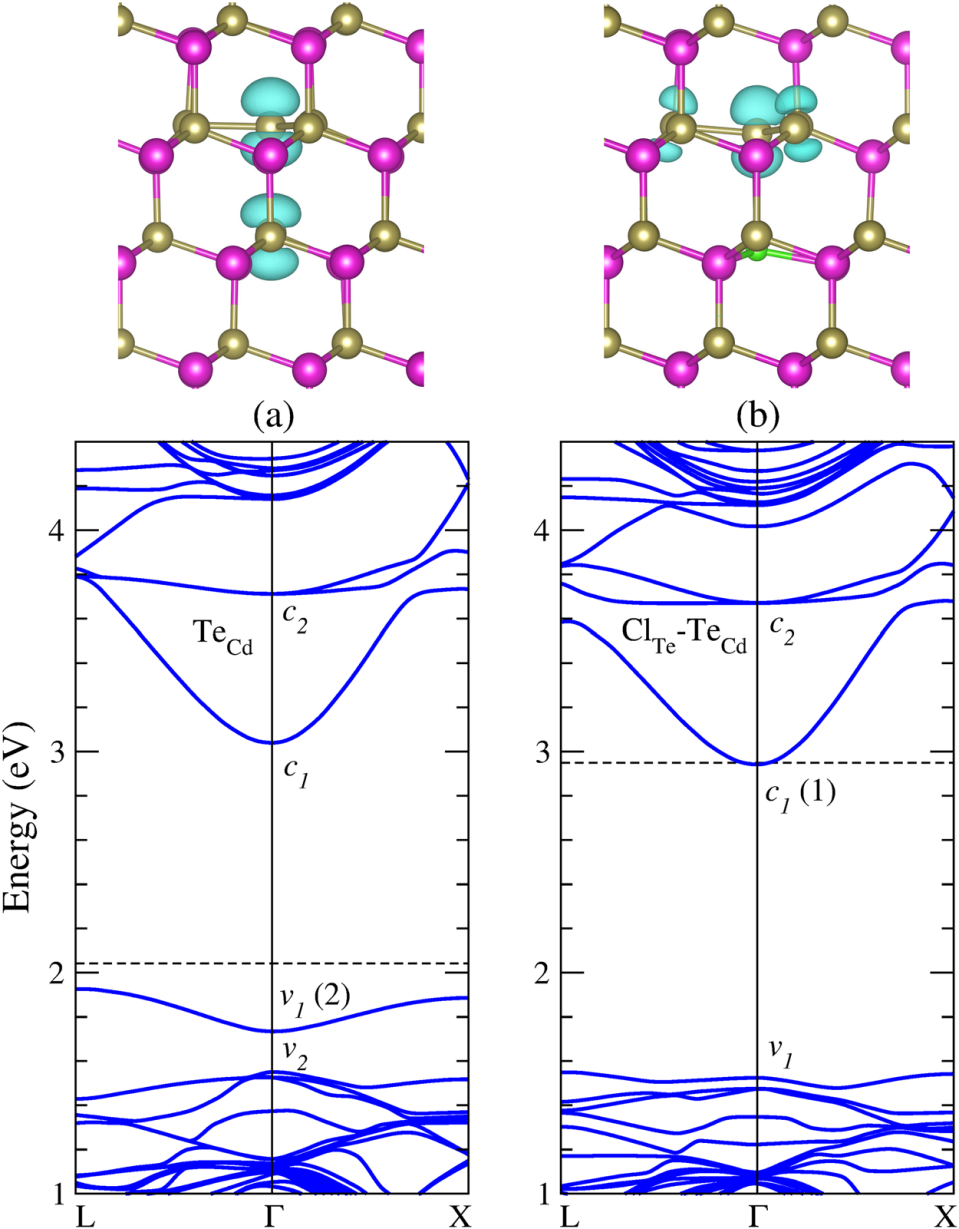
Equilibrium geometry and band structure calculations of (**a**) the Te_Cd_ antisite, and (**b**) the Cl_Te_-Te_Cd_ complex, in the neutral charge state. The dashed line indicates the Fermi energy. Charge density isosurfaces of the occupied level in the band gap (*v*_1_) in (**a**) and the VBM level (*v*_1_) in (**b**) are plotted for *ρ* = 0.005 *e*/Å^3^ and *ρ* = 0.001 *e*/Å^3^, respectively.

We now turn to examine the complex formed by the substitutional chlorine (Cl_Te_) nearest neighbor to the Te antisite (Te_Cd_). Previous DFT calculations have extensively studied the Te_Cd_ antisite because is one of the most abundant native defect under Te-rich growth conditions^7,35,40,48–52^. Lindström *et al*.^52^ using the hybrid functional HSE06 have reported three charge states for this defect: ${{\rm{Te}}}_{{\rm{Cd}}}^{2+}$, ${{\rm{Te}}}_{{\rm{Cd}}}^{0}$, and ${{\rm{Te}}}_{{\rm{Cd}}}^{2-}$, which are stable in *T*_*d*_, *C*_3*v*_ and *C*_*s*_ symmetries, respectively. We found similar results, but according to our formation energy calculations only ${{\rm{Te}}}_{{\rm{Cd}}}^{2+}$ and ${{\rm{Te}}}_{{\rm{Cd}}}^{0}$ would be stable, as shown in Fig. 8. The discrepancy can be attributed to finite size effect, as the authors of ref.^52^ applied a potential alignment scheme in a 128-atom supercell, in contrast to our formation energy calculations performed with a larger 250-atom supercell. It is interesting to note that Te_Cd_ shows higher formation energies than Cl_Te_ for all values of the Fermi energy in the band gap, as can be seen by comparing Figs 8 and 2, suggesting that the chlorine impurity would be energetically favorable over any native defect. Moreover, the Te_Cd_ defect exhibits a negative-U behavior, where the single-positive charge state is never stable, in agreement with previous calculations^40,50,52,53^. Hence, the Te_Cd_ defect shows a *ε*(2 + /0) transition level at VBM + 0.4 eV. However, in a previous work using accurate quasiparticle DFT + GW calculations we found this state at VBM + 1.0 eV^53^. The electronic structure of ${{\rm{Te}}}_{{\rm{Cd}}}^{2+}$ shows an empty *t*_2_ level in the higher part of the CdTe band gap. After capturing two electrons, the defect experiences a Jahn-Teller distortion, which is characterized by the breaking a Te-Te bond, lowering the symmetry from *T*_*d*_ to *C*_3*v*_. In this way, the *t*_2_ level split into a fully-occupied *a*_1_ level and an empty *e* level, which are represented by *v*_1_ and *c*_2_ levels in the ${{\rm{Te}}}_{{\rm{Cd}}}^{0}$ band structure, shown in Fig. 9(a).

Concerning Cl_Te_-Te_Cd_, our results suggests that this complex only exists in single positive charge state with similar formation energy than the Te_Cd_ antisite, as shown Fig. 8. Figure 9(b) shows the band structure calculations of this complex, indicating a shallow donor character, without exhibiting deep levels in the band gap. This result suggests the passivation of the fully-occupied *a*_1_ level of ${{\rm{Te}}}_{{\rm{Cd}}}^{0}$ after the formation of the Cl_Te_-Te_Cd_ complex, providing an additional explanation to the beneficial effect of the chlorine treatment as experimentally reported.

### Interstitial chlorine in CdTe

In an earlier paper^54^, we discussed the role of interstitial chlorine in CdTe (Cl_*i*_). In that study we found that Cl_*i*_ is stable in at least five distinct interstitial sites with close formation energies. Moreover, this impurity introduces shallow energy levels that may lead to a donor-acceptor compensation mechanism. Nevertheless, the dominant configurations of Cl_*i*_ have a formation energies higher than Cl_Te_ and the 2Cl_Te_-V_Cd_ complex for values of the Fermi level near midgap, under both Te-rich and Cd-rich growth conditions. We refer the interested reader to ref.^54^, the work of Lindström *et al*.^36^, and the topical review of  Yang *et al*.^44^.

### Self-compensation in chlorine-doped CdTe

**Figure 10:**
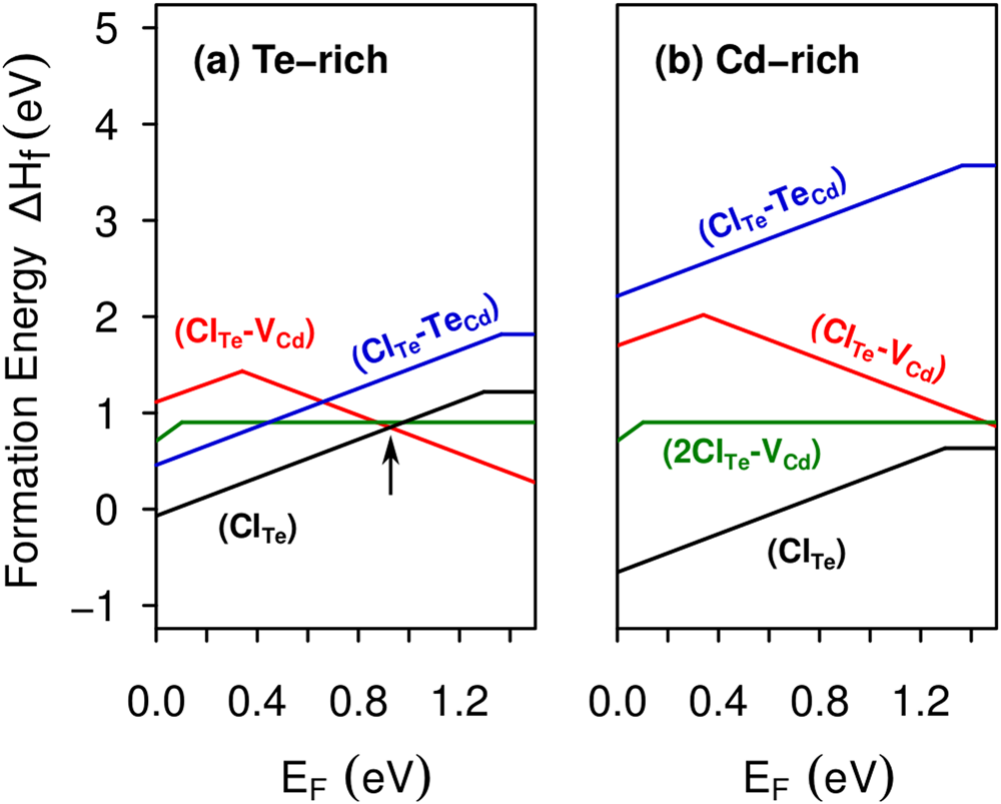
Formation energies of most stable Cl-related defects in CdTe, as a function of the Fermi level, under (**a**) Te-rich condition and (**b**) Cd-rich condition.

Figure 10 summarizes the formation energies Cl-related defects in CdTe, which are likley to be formed due to their low formation energies. For values of the Fermi level in the lower part of the band gap (*n*-type CdTe), we observe that (Cl_Te_)^+^ is the most relevant defect under both Te-rich and Cd-rich growth conditions, acting as the dominant donor. Whereas, for the Fermi level in the higher part of the band gap (*p*-type CdTe), the relevant defect is the complex [(Cl_Te_-V_Cd_)(*C*_3*v*_)]^−^, which is the dominant acceptor. Therefore, in the Te-rich limit, the closed-shell (Cl_Te_)^+^ donor would be compensated by the [(Cl_Te_-V_Cd_)(*C*_3*v*_)]^−^ acceptor after transferring its electron in excess, leading to the Fermi level pinning at VBM + 0.92 eV, as indicate the arrow in Fig. 10(a).

Interestingly, at the crossing point between (Cl_Te_)^+^ and [(Cl_Te_-V_Cd_)(*C*_3*v*_)]^−^ in Te-rich CdTe, the complex 2Cl_Te_-V_Cd_ is only 0.05 eV higher in energy [see Fig. 10(a)]. Thus, the existence of 2Cl_Te_-V_Cd_ complexes will contribute to stabilize the charge neutrality condition, allowing the Fermi level pinning in CdTe:Cl without introducing a compensating deep level at variance of the cases of CdTe:Sn^27^ and CdTe:Ge^55^. These results are in good agreement with the earlier work of Höschl *et al*.^56^, who suggested that Cl_Te_-V_Cd_ and 2Cl_Te_-V_Cd_ dominate (59% and 40%) over Cd vacancies as compensating acceptors in semi-insulating CdTe:Cl. Moreover, Lindström *et al*.^36^ also suggested the possible formation of a tricomplex consisting of two Cl ions and one Cd vacancy. An alternative explanation for the deep level observed by spectroscopy methods^34^ could be the possible formation of chlorine complexes with Te antisites, which are the dominant recombination centers in CdTe^53^. However, our results for the Cl_Te_-Te_Cd_ complex do not show any deep levels in the band gap. In addition, our calculations show that Cl_Te_ is the dominant defect in the Cd-rich limit for any value of the Fermi energy, suggesting that chlorine can be used as an effective *n*-type dopant under these conditions. For instance, the incorporation of Cl under controlled Cd partial pressures might help to populate the intermediate band in CdTe:Sn^27^.

## Summary

In summary, we have investigated the electronic structure, formation energies, and transition states of substitutional chlorine Cl_Te_ and Cl_Cd_, and the complexes formed by Cl_Te_ with the most probable defects found in Te-rich CdTe, namely the Cd vacancy and the Te antisite. We find that the Cl_Te_-V_Cd_ complex can exist in two geometries, with *C*_*s*_ and *C*_3*v*_ symmetries, showing shallow donor and acceptor properties, respectively. We also identify a second complex containing two substitutional chlorine neighboring to the cadmium vacancy 2Cl_Te_-V_Cd_, which also can exist in two geometries, both with *C*_2*v*_ symmetry. The latter is found stable only in double positive charge state for *n*-type CdTe, while the former exhibits a ground-state configuration. Concerning the Cl_Te_-Te_Cd_ complex, our results suggest that it is stable only in single positive charge state with similar formation energy than the Te_Cd_ antisite. For a Fermi energy close to the middle of the CdTe band gap, the three complexes show formation energies of around 1 eV under Te-rich condition, as shown in Fig. 10(a), suggesting that they are equally likely to be found. Table 1 compares the formation energy values for all the Cl-related defects studied under Te-rich and Cd-rich conditions for the Fermi energy at the VBM.

**Table 1:** Formation energy (in eV) of the Cl-related defects in CdTe under study. The Fermi energy is set at the valence band maximum. Lower values for the formation energy, in general less than 1 eV, indicates the most likely defects to be found.

Defect	Δ*Hf* (Te rich)	Δ*Hf* (Cd rich)
(Cl_Te_)^1+^	−0.07	−0.65
(Cl_Te_)^0^	1.22	0.63
(Cl_Cd_)^1−^	2.92	4.68
(Cl_Cd_)^0^	2.82	4.57
[(Cl_Te_-V_Cd_) (*C*_*s*_)]^1+^	1.11	1.70
[(Cl_Te_-V_Cd_) (*C*_*s*_)]^0^	2.42	3.00
[(Cl_Te_-V_Cd_) (*C*_3*v*_)]^1−^	1.78	2.36
[(Cl_Te_-V_Cd_) (*C*_3*v*_)]^0^	1.68	2.27
[(2Cl_Te_-V_Cd_) (*d*)]^2+^	0.71	0.71
[(2Cl_Te_-V_Cd_) (*d*)]^1+^	1.81	1.81
[(2Cl_Te_-V_Cd_) (*d*)]^0^	3.06	3.06
[(2Cl_Te_-V_Cd_)]^0^	0.90	0.90
(Te_Cd_)^2+^	0.53	2.87
(Te_Cd_)^0^	1.29	3.63
(Cl_Te_-Te_Cd_)^1+^	0.46	2.21
(Cl_Te_-Te_Cd_)^0^	1.82	3.57

Finally, we find that neither the complexes under study nor the substitutional Cl impurity induce deep level in the CdTe band gap. Therefore, our calculations suggest that self compensation between Cl-induced shallow donors and acceptors should be responsible for the high resistivity observed in detector-grade CdTe:Cl, usually grown in a Te-rich environment, in agreement with previous hypothesis^35^. Particularly, the (Cl_Te_)^+^ shallow donor would be compensated by the [(Cl_Te_-V_Cd_)(*C*_3*v*_)]^−^ shallow acceptor, leading to the Fermi level pinning at VBM + 0.92 eV. In addition, our results show that the formation of the Cl_Te_-Te_Cd_ complex passivates the deep level associated to the Te antisite (Te_Cd_), confirming to some extent the beneficial effect of chlorine in CdTe.

## Methods

We performed first-principles calculations based on the density functional theory (DFT)^57,58^, as implemented in the Vienna Ab Initio Simulation Package (VASP)^59^. We used the Heyd-Scuseria-Ernzerhof (HSE06)^60,61^ hybrid functional with the standard mixing parameter *α* = 25%, a plane-wave basis set with a cutoff energy of 285 eV. The core-valence interaction is described by the projector augmented-wave mathod^62^. Our calculations were performed using large 250-atom supercells, which were fully relaxed until the forces on each atom were less than 0.025 eV/Å. Our computational approach improves previous theoretical works in which smaller 64-atom supercell were used^36,44,63^, despite the fact that the convergence of hybrid functionals with respect to the supercell size may be slower than its local and semi-local counterparts^15,64,65^. Additionally, due to the high computational cost of the HSE06 calculations, only the Γ point was used for the Brillouin zone sampling, whereas band structures calculations were performed using a 128-atom supercell.

The formation energy (Δ*H*_*f*_) of a defect in charge state *q* can be written as a function of the Fermi level (*E*_F_) and the chemical potentials of the atomic species (*μ*_*i*_) as follows^66^:

1 $${\rm{\Delta }}{H}_{f}[{E}_{F}]={E}_{tot}[{X}^{q}]-{E}_{tot}[{\rm{bulk}}]-\sum _{i}{n}_{i}{\mu }_{i}+q({E}_{F}+{E}_{{\rm{VBM}}}),$$

where *E*_*tot*_[*X*^*q*^] and *E*_*tot*_[bulk] are the total energy of the system with a defect in charge state *q* and the pristine system, respectively. *E*_VBM_ is the energy of the valence band maximum. *n*_*i*_ is the number of atoms of type *i* that have added or removed from a pristine system. For instance, in the case of Cl substituting a Cd atom in the charge state *q*, the formation energy can be obtained as:

2 $${\rm{\Delta }}{H}_{f}[{E}_{F}]={E}_{tot}[{({{\rm{Cd}}}_{n-1}{{\rm{Te}}}_{n}{\rm{Cl}})}^{q}]-{E}_{tot}[{{\rm{Cd}}}_{n}{{\rm{Te}}}_{n}]+{\mu }_{{\rm{Cd}}}-{\mu }_{{\rm{Cl}}}+q({E}_{F}+{E}_{{\rm{VBM}}}),$$

where *E*_*tot*_(Cd_*n*_Te_*n*_) is the total energy of a supercell containing *n* primitive cells of CdTe and *E*_*tot*_(Cd_*n*−1_Te_*n*_Cl)^*q*^ is the energy of the same supercell with one Cd atom replaced by a Cl atom with *q* electrons removed. For Cl substituting a Te atom in charge state *q* (${{\rm{Cl}}}_{{\rm{Te}}}^{q}$), the same procedure is applied, just exchanging Te by Cd in Eq. (2).

The chemical potentials in Eq. (2) are defined as:

3 $${\mu }_{{\rm{X}}}={E}_{{\rm{X}}}+{\rm{\Delta }}{\mu }_{{\rm{X}}},\,{\rm{with}}\,{\rm{X}}=\{{\rm{Cd}},\,{\rm{Te}},\,{\rm{Cl}}\},$$

where *E*_X_ is the energy per atom of bulk-phase Cd and Te, and gas-phase Cl (Cl_2_). In addition, the thermodynamic equilibrium requires the following restrictions to the chemical potentials:

4 $${\rm{\Delta }}{\mu }_{{\rm{Cd}}}\le \mathrm{0,}\,{\rm{\Delta }}{\mu }_{{\rm{Te}}}\le \mathrm{0,}\,{\rm{\Delta }}{\mu }_{{\rm{Cl}}}\le \mathrm{0,}$$

5 $$2{\rm{\Delta }}{\mu }_{{\rm{Cl}}}+{\rm{\Delta }}{\mu }_{{\rm{Cd}}}\le {\rm{\Delta }}H({{\rm{CdCl}}}_{2})=-\,\,3.59\,{\rm{eV}},$$

6 $${\rm{\Delta }}{\mu }_{{\rm{Cd}}}+{\rm{\Delta }}{\mu }_{{\rm{Te}}}={\rm{\Delta }}H({\rm{CdTe}})=-\,\,1.17\,{\rm{eV}}.$$

Inequalities (4) and (5) represent necessary conditions to avoid that elements and compounds segregate, whereas Δ*μ*_Cd_ = 0 and Δ*μ*_Te_ = 0 represent Cd-rich and Te-rich conditions, respectively. Δ*H*(C*dCl*_2_) and Δ*H*(CdTe) are the heats of formation of CdCl_2_ and CdTe. The numerical values of heats of formation are obtained from HSE06 calculations. Relations (4) and (6) impose that both Δ*μ*_Cd_ and Δ*μ*_Te_ are larger than Δ*H*(CdTe). Others expressions like (5) can be set to avoid the formation of other compounds like TeCl_4_ and Te_3_Cl_2_, but in practice (5) sets the maximum possible value for Δ*μ*_Cl_ in equilibrium with CdTe. *E*_*tot*_(Cd_*n*_Te_*n*_Cl)^*q*^ in Eq. (2) includes size corrections for electrostatic interactions between nearest-neighbor images in the supercell calculation^67^. No additional corrections for band filling are needed^67^, as we used only the Γ point for the Brillouin zone sampling.
